# MIF: Implications in the Pathoetiology of Systemic Lupus Erythematosus

**DOI:** 10.3389/fimmu.2015.00577

**Published:** 2015-11-11

**Authors:** Tali Lang, Andrew Foote, Jacinta P. W. Lee, Eric F. Morand, James Harris

**Affiliations:** ^1^Lupus Research Group, Monash Centre for Inflammatory Diseases, School of Clinical Sciences at Monash Health, Faculty of Medicine, Nursing and Health Sciences, Monash Medical Centre, Clayton, VIC, Australia

**Keywords:** MIF, SLE, therapeutics, autophagy, innate immunity and responses

## Abstract

Macrophage migration Inhibitory factor (MIF) was one of the earliest pro-inflammatory cytokines to be identified. Increasing interest in this cytokine in recent decades has followed the cloning of human MIF and the generation of Mif^−/−^ mice. Deepening understanding of signaling pathways utilized by MIF and putative receptor mechanisms have followed. MIF is distinct from all other cytokines by virtue of its unique induction by and counter regulation of glucocorticoids (GCs). MIF is further differentiated from other cytokines by its structural homology to specific tautomerase and isomerase enzymes and correlative *in vitro* enzymatic functions. The role of MIF in immune and inflammatory states, including a range of human autoimmune diseases, is now well established, as are the relationships between MIF polymorphisms and a number of inflammatory diseases. Here, we review the known pleiotropic activities of MIF, in addition to novel functions of MIF in processes including autophagy and autophagic cell death. In addition, recent developments in the understanding of the role of MIF in systemic lupus erythematosus (SLE) are reviewed. Finally, we discuss the potential application of anti-MIF strategies to treat human diseases such as SLE, which will require a comprehensive understanding of the unique and complex activities of this ubiquitously expressed cytokine.

## Introduction

Macrophage migration inhibitory factor (MIF) also known as glycosylation-inhibiting factor (GIF) is a multifunctional protein with a broad range of immunomodulatory properties. The existence of a MIF has been hypothesized since the experiments of Rich and Lewis (1), where it was shown that tuberculin-induced delayed-type hypersensitivity reactions (DTH) were associated with the inhibition of macrophage migration. However, it was not until 1966 that MIF was first described as a soluble factor responsible for the inhibition of emigration of macrophages during DTH (2, 3). MIF acts as a mediator of innate immunity by promoting host inflammatory responses through induction of pro-inflammatory cytokines, including TNF-α and IL-6. MIF can also modulate host inflammatory responses by regulating cellular processes such as T-cell proliferation, suppression of p53-dependent apoptosis, and counter regulation of the immunosuppressive actions of glucocorticoids (GCs). Human MIF cDNA was first isolated in 1989 (4), although both human and murine forms of MIF were not cloned and functionally tested until the early 1990s by Bernhagen and colleagues (5–7). The first MIF knockout mice (Mif^−/−^), reported in 1999, were generated through disruption and deletion of exon 3 in the MIF gene (8). Since then there has been significant scientific interest in MIF, which has been shown to function not only as a pro-inflammatory protein but also as a stress factor and a growth factor (9) released by cells of the anterior pituitary gland, similar to a hormone (10).

## MIF Genetics and Protein Structure

Migration inhibitory factor is a non-glycosylated 12.5-kDa protein composed of 114 amino acids, highly conserved across species with murine MIF showing 90% homology to human MIF (11). Within the human genome, the *MIF* gene is located on chromosome 22 (22q11.23). MIF is composed of three short exons of 107, 172, and 66 bp and two introns of 188 and 94 bp (11). Crystal structures demonstrate that MIF is a homotrimer with structural homology to three bacterial enzymes; oxalocrotonate tautomerase, 5-carboxymethyl-2-hydroxymuconate isomerase, and chorismate mutase (12–16). Within recent years, a gene homologous to *MIF*, which encodes the protein *d-dopachrome tautamerase* (*D-DT*), has been included in the MIF superfamily (17). *MIF* and *D-DT* are located within close proximity on chromosome 22, and are nearly identical in exon lengths with variable non-coding intron regions.

*MIF* and *D-DT* gene expression are both regulated by transcription factors. MIF is regulated by ten known polymorphic sites, as previously described within the *MIF* gene (18). Two of these polymorphisms have been demonstrated to have functional impact and to influence susceptibility to and/or severity of a number of diseases (Table 1). The first is a short-tandem repeat (STR), which is a microsatellite repetition consisting of cytosine–adenine–thymine–thymine (CATT) at position −794 bp, −794 CATT_5−8_ (rs5844572) within the 5′ promoter region (19). High expression alleles such as −794 CATT_7_ have been associated with an increase in *MIF* gene expression, increased levels of circulating MIF (20), and severity in clinical phenotypes (20). Conversely, the sub-Saharan, low expression −794 CATT_5_ allele is associated with reduced levels of circulating MIF (21). The second polymorphism is a single nucleotide polymorphism (SNP) in which guanine (G) is replaced with cytosine (C) in the *MIF* gene at position −173 bp, −173 G > C (rs755662) (22). The −173*C allele has also been shown to correlate with increased levels of circulating MIF, as identified in several populations (20, 23). Based on findings published to date, it can be postulated that *MIF* promoter polymorphisms and consequent changes in MIF expression contribute to the susceptibility and clinical severity of many inflammatory and autoimmune disorders where MIF has been implicated. However, one should be cautious about associations made between expression of *MIF* alleles and clinical severity and/or susceptibility, and study limitations, such as ethnic populations recruited as well as overall cohort size, which may influence outcomes in gene association studies due to population stratification of the *MIF* gene locus, need to be considered.

**Table 1:** Associations between MIF −173*C and −794 CATT_5−__8_ polymorphisms and autoimmune disease.

Disease	MIF polymorphism	Effect	Reference
Rheumatoid arthritis	−794 CATT_5_	Protective	(19)
	−794 CATT_7_, −173C	Increased severity, radiological progression	(20)
	−794 CATT_7_, −173C	Do not predict response to glucocorticoid treatment or anti-TNF- α therapy	(24)
	−173C	Increased susceptibility amongst CRP-negative patients	(25)
	−173C	Increased susceptibility (meta analysis)	(26)
	794 CATT_7_, −173C	Associated with early onset, associated with high disease activity	(27)
Juvenile idiopathic arthritis	−173C, −794 CATT_7_, −173C haplotype	Increased susceptibility	(22, 23)
		Increased susceptibility	(28)
	−173C	Increased susceptibility (meta analysis)	(29)
	−173C	No link to susceptibility but strong predictor of poor prognosis	(30, 31)
	−173C	Predictor of poor response to glucocorticoids	(32)
Inflammatory polyarthritis	−173C, −794 CATT_7_	Increased susceptibility, but no link to severity	(33)
Rheumatic fever	−173C	Increased susceptibility	(34)
Systemic lupus erythematosus	−173C, −794 CATT_7_, −794 CATT_7_−173C haplotype	Increased susceptibility, Increased severity, Increased TNF-α	(35, 36)
	−173C, −794 CATT_7_	Reduced susceptibility	(37)
	−794 CATT_5_	Protective against tissue damage	(37)
Psoriasis	−173C, −174 CATT7, −794 CATT_7_ −173C haplotype	Increased susceptibility	(38, 39)

Ulcerative colitis (UC)	−173C	Increased susceptibility	(40–44)
	−173C	No association	(45, 46)
	−173C	Increased pancolitis	(47)
	−794 CATT_7_	Increased susceptibility and severity	(48)
	−794 CATT_5_	Protective	(48)
Crohn’s disease (CD)	−173C	Protective	(45, 49)
	−173C	No effect	(43)
Celiac disease	−173C, −794 CATT_7_, −794 CATT_7_−173C haplotype	Increased susceptibility	(50)

## Signal Transduction and Regulation of Cell Activation

**Figure 1:**
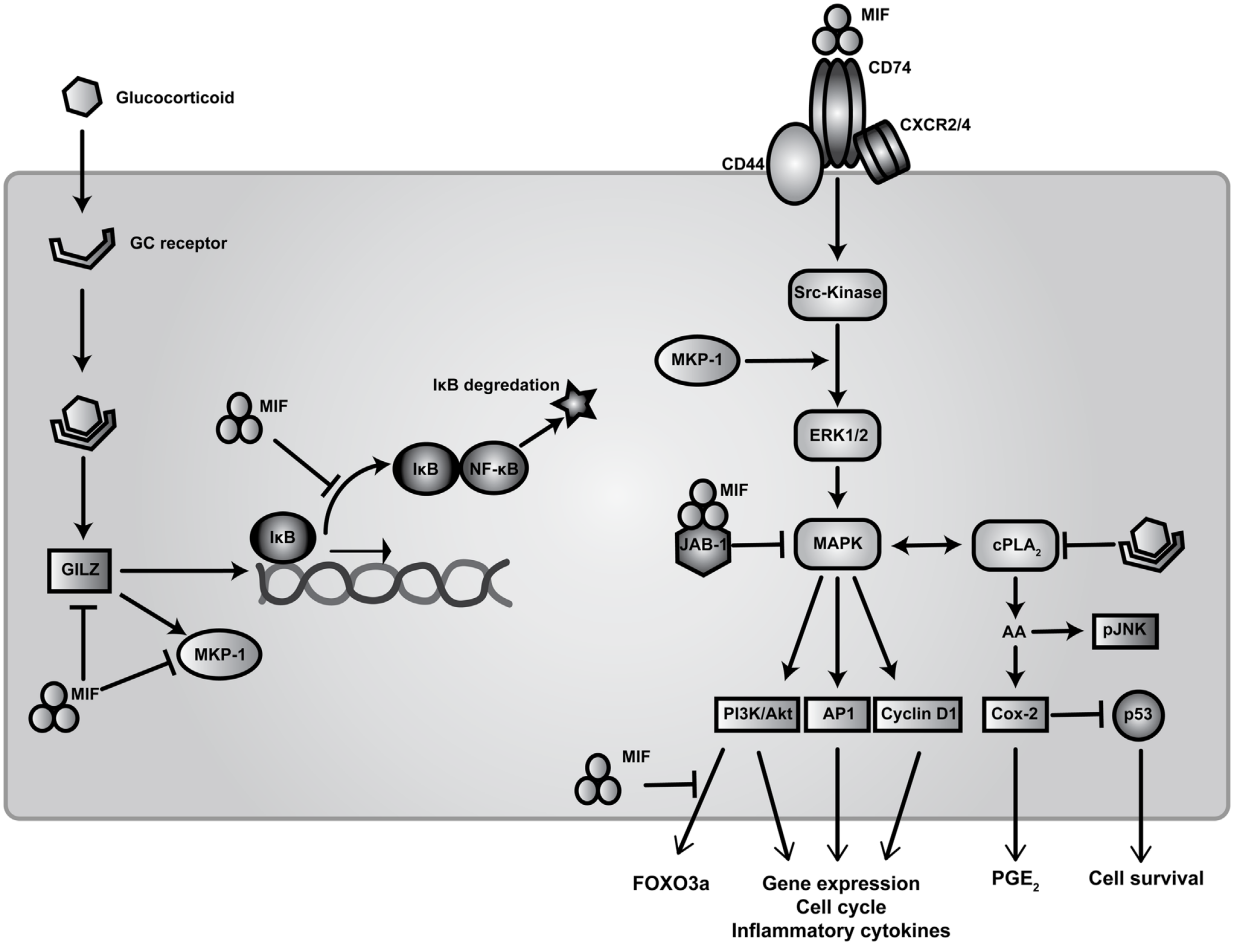
**Signal transduction pathways of MIF and regulation of glucocorticoid immunosuppression**. Extracellular MIF binds to the transmembrane receptor complex CD74 and CD44 to activate downstream Src-family kinase resulting in the subsequent phosphorylation of ERK/MAPK. This facilitates activation of transcription elements AP1, PI3K/Akt, and Cyclin D1 leading to expression of pro-inflammatory cytokines, cell cycle regulators, and co-stimulatory genes. MIF also binds to G-protein-coupled chemokine receptors CXCR2 and CXCR4 to promote calcium influx and integrin activation. Following endocytosis, MIF interaction with JAB-1 down-regulates MAPK thereby modulating cellular redox homeostasis. Elevated levels of MIF inhibit p53-mediated apoptosis through enhanced _c_PLA_2_ activity resulting in increased AA production and PGE_2_ release. Intracellular AA is required for the activation of pJNK for transcriptional stability of mRNAs for TNF-α and other cytokines. These signaling events can be inhibited by GCs via modulation of _c_PLA_2_ activity. GCs prevent expression of NF-ĸB-dependent genes primarily by increasing expression of IĸB. GC induced expression of MKP-1, which inactivates MAPK activity in response to pro-inflammatory stimuli, is inhibited by MIF through the dephosphorylation of multiple MAPK members. Finally, MIF can counter-regulate the expression of both MKP-1 and GILZ through blocking Akt-dependent activation and translocation of FoxO3a (55).

Migration inhibitory factor is a highly pleiotropic cytokine, as reflected by the complexity of its involvement in regulating multiple signal transduction pathways (Figure 1). Cellular activation by MIF is reportedly initiated through interactions with its proposed receptor, CD74 – the cell surface form of the MHC class II invariant chain – which subsequently forms a signaling complex with the accessory protein CD44 (51). MIF has also been reported to interact with the chemokine receptors CXCR2 and CXCR4 in complexes involving CD74 (52–54).

Migration inhibitory factor activates Src-family tyrosine kinases downstream of extracellular signal regulated kinase (ERK1/2) and p38, both members of the mitogen-activated protein kinase (MAPK) family (56, 57). Sustained activation of ERK is attained via c-Jun activation domain binding protein (JAB-1) (58). ERK activation leads to the phosphorylation of cytosolic proteins as well as up-regulation of phospholipase A_2_ (_c_PLA_2_) activity to produce prostaglandin precursors from arachidonic acid (AA) (56). AA activates JUN-terminal kinase (JNK), which is in turn required for activation of downstream regulatory elements ETS, PI3K/Akt, and AP1 (56, 59). These pathways lead to the transcription of pro-inflammatory cytokines, such as TNF-α, as well as chemokines such as CCL2 (MCP-1), implicated in the development of many autoimmune diseases, including SLE (60).

Elevated levels of MIF also result in reduced p53 accumulation in the cytoplasm, thereby blocking p53-mediated cell death and leading to continuous production of pro-inflammatory cytokines such as TNF-α, IL-1β, IL-6, and prostaglandins (11, 56, 61). This process occurs in an autocrine manner, whereby MIF phosphorylates ERK1/2 and activates _c_PLA_2_ and cyclooxygenase-2 (Cox-2), blocking p53-induced apoptosis. MIF has also been shown to counter-regulate GC-induced expression of MAPK phosphatase-1 (MKP-1), a critical MAPK signaling inhibitor, through which GCs signal to suppress pro-inflammatory cytokine secretion (62, 63). It was shown by Roger and others that MIF targets MKP-1 in an autocrine manner to prevent GC-induced MKP-1 expression, thereby tempering the post-transcriptional inhibition of cytokine production by GCs (62, 64). Moreover, MIF-deficient macrophages show increased sensitivity to GCs following LPS stimulation, with higher levels of MKP-1 expression and reduced activation and phosphorylation of p38-MAPK (62, 64). However, in this study no effect of GCs on IĸBα levels was observed. Correspondingly, there was no interference in the ability of NF-ĸB to translocate to the nucleus and bind the TNF-α promoter, which correlates with previous findings from earlier studies (63, 65, 66). It has been proposed that decreased levels of MIF leads to increased MKP-1, resulting in destabilization of AU-rich elements found in mRNA of multiple cytokines, via phosphorylation and activation of the downstream target MAPK-activated protein kinase-2 (MAPKAPK2) (67). Therefore, MIF counter regulation of anti-inflammatory actions by GCs results in the reduced expression of MKP-1, increased activity of p38 and MAPKAPK2 and greater stability of mRNAs conferring AU-rich element-dependent translation.

Studies from own lab have identified a novel molecular mechanism through which MIF can regulate the expression of MKP-1 and activation of MAPK through the GC-responsive protein, GC-induced leucine zipper (GILZ, also known as TSC22 domain family protein 3) (68). GILZ interacts with numerous signaling pathways relevant to inflammatory diseases (69). We recently demonstrated that exogenous MIF inhibited both GILZ and MKP-1 expression in fibroblasts and macrophages. MIF regulation of GILZ was shown to occur through inhibition of the Akt-dependent nuclear translocation of the transcription factor, FoxO3a. Moreover, MIF inhibition of MKP-1 expression was dependent on this inhibition of GILZ, suggesting a novel mechanism through which MIF impairs GC sensitivity (17).

## MIF and Immunity

Migration inhibitory factor is produced by most cells of the immune system, including monocytes, macrophages, blood dendritic cells, B-cells, T-cells, neutrophils, eosinophils, mast cells, and basophils (1). MIF is constitutively expressed and stored in intracellular pools, so does not require *de novo* synthesis for secretion. MIF is secreted by macrophages following stimulation with LPS, or other pro-inflammatory cytokines such as TNF and IFN-γ (6). Moreover, macrophage-derived MIF can stimulate the synthesis of other pro-inflammatory mediators via autocrine and paracrine effects, enhancing macrophage functions, including phagocytosis, reactive oxygen species (ROS) production, and nitric oxide (NO) production (6, 70–72).

Migration inhibitory factor is secreted by pituitary cells following LPS stimulation *in vivo* and this contributes significantly to circulating MIF in the post-acute phase of LPS-induced endotoxemia. Furthermore, co-injection of MIF with LPS increases lethality, while anti-MIF antibody protects mice against LPS-induced endotoxemia (10). The small molecule MIF antagonist, ISO-1 ((S,R)-3-(4-hydroxyphenyl)-4,5-dihydro-5-isoxazole acetic acid methyl ester), also protects mice against LPS-induced endotoxemia and reduces TNF-α production by peritoneal macrophages (73). Similarly, MIF-deficient mice are protected against lethal sepsis induced by LPS or *Staphylococcus aureus* enterotoxin B (SEB) with d-galactosamine (8).

While MIF clearly has pathogenic roles to play in responses to bacterial products, it also facilitates the detection of endotoxin-containing bacteria through the up-regulation of TLR4 in macrophages, allowing rapid and protective pro-inflammatory responses to these pathogens (11). Consistent with this, MIF-deficient mice were more susceptible to infection with *Salmonella typhimurium*, producing lower levels of IL-12, IFN-γ, and TNF-α (74). More recently, MIF has been shown to play a role in protection against *Mycobacterium tuberculosis*, which does not express LPS and has a more complex relationship with TLR4 (75). Mif^−/−^ mice are more susceptible to infection with either *M. tuberculosis* or *Mycobacterium bovis* BCG and demonstrate inhibited secretion of TNF-α, IL-12, and IL-10 (76). Moreover, the low expresser *MIF* genotype −794 CATT_5/5_ is enriched in a cohort of Ugandan patients with HIV and disseminated tuberculosis (TB) (76).

Migration inhibitory factor is constitutively expressed by T cells and secreted in response to mitogenic or antigenic stimulation (77, 78); treatment with anti-MIF antibody reduces T cell IL-2 production and proliferation (78–81). *In vivo*, MIF has a well-defined role in DTH, which is inhibited by MIF neutralization or deficiency, leading to decreased antigen-specific T cell proliferation, IgG production and IFN-γ secretion (78, 80, 82). MIF can stimulate secretion of both Th1 and Th2 cytokines by T cells (78), as well as IL-17 by lymph node cells (83), suggesting no single clear role in T cell polarization. MIF can also facilitate leukocyte recruitment and trafficking through the up-regulation of classical chemokines, such as CXCL8 (IL-8), CCL-5 (RANTES), and CCL-2 (MCP-1) (57, 84, 85). Moreover, MIF is now suggested to be a non-cognate ligand of the chemokine receptors CXCR2 and CXCR4, through which it can influence chemotaxis of monocytes, T cells, and B cells directly (52, 86). Interestingly, in the context of tumor immunology it has been demonstrated that MIF promotes the infiltration of immune-suppressive cells, including myeloid-derived suppressor cells (MDSCs) and Tregs (87, 88). These cell sub-populations have been implicated in tumor progression and metastasis by limiting anti-tumor immunity, as well as inducing immune tolerance. It is unknown whether MIF similarly modulates immune-suppressive cells in the context of other inflammatory diseases, including SLE.

## MIF and Autophagy

Autophagy is a catabolic pathway for the delivery of cytosolic constituents, including long-lived proteins, protein aggregates and organelles, to lysosomes for degradation. Activated during nutrient deprivation, autophagy acts as a cytoprotective mechanism for amino acid recycling (89). In addition, autophagy has been shown by many groups to regulate the transcription, processing and secretion of pro-inflammatory cytokines (90). Inhibition of autophagy increases the secretion of IL-1α, IL-1β, IL-18, and IL-23 by macrophages and dendritic cells in response to TLR agonists (91–94). This process is dependent on the accumulation of ROS and mitochondrial DNA in the cytosol (95). Conversely, induction of autophagy inhibits the secretion of IL-1β and IL-23 (92, 96). Recent studies have demonstrated that MIF can regulate autophagy. In one study, MIF was shown to suppress a phenomenon termed autophagic cell death in the human MCF-7 breast cancer cell line (97). This is likely due to activation of the PI3K/Akt pathway, which inhibits autophagy (98, 99). In contrast, other studies have suggested that MIF induces or facilitates autophagy in mouse myoblasts, cardiomyocytes and human HuH-7 hepatoma cells (100–103). MIF-induced autophagy in HuH-7 cells was dependent on the generation of ROS and, interestingly, starvation induced MIF secretion, again dependent on ROS (100). However, it is not clear whether this is an autophagy-dependent process, or a side effect of amino acid starvation, independent of autophagy induction.

Given that autophagy has been linked to a number of inflammatory diseases (104) and there is evidence to suggest that autophagy is dysregulated in SLE patients (105–108), a better understanding of how MIF intersects with this important cellular process could prove highly significant.

## MIF and Systemic Lupus Erythematosus

Given its pleiotropic role in the regulation of inflammatory cytokines and leukocyte trafficking, it is perhaps unsurprising that MIF has been linked with a number of autoimmune and inflammatory diseases. Genetic studies have identified associations between *MIF* polymorphisms and autoimmune diseases, including rheumatoid arthritis (RA), systemic lupus erythematosus (SLE), type I diabetes, and autoimmune liver disease (Table 1). Here, we will discuss the association of *MIF* polymorphisms and their relevance to disease progression, severity and clinical outcomes in SLE.

Systemic lupus erythematosus is complex chronic multi-organ autoimmune disease of unknown etiology with significant heterogeneity in clinical manifestations. It is generally considered a multifactorial disease, as a combination of genetics, environmental triggers, sex hormones, and other factors are thought to be involved (109). SLE is most prevalent within African-American and Asian populations, typically in females of childbearing age (110, 111). SLE is characterized by loss of tolerance to nucleic acids and their interacting proteins, resulting in the development of autoantibodies, inflammation, and tissue damage (109). Our own lab has demonstrated that levels of circulating MIF are raised in patients with SLE and are positively associated with disease damage (measured by SLICC/ACR index) and, interestingly, GC use (112). Similarly, renal MIF is increased in patients with lupus (and non-lupus) proliferative glomerulonephritis, correlating with leukocyte infiltration, tissue damage and impairment of renal function (113). To date, it is unknown whether kidney injury associated with SLE contributes to elevated serum MIF, and thus future studies examining levels of serum and urine MIF in relation to lupus nephritis are needed.

In the lupus-prone MRL/*lpr* mouse strain, MIF expression has been demonstrated to be increased in both skin and kidney lesions. Correspondingly, Mif^−/−^ mice showed prolonged survival, reduced renal and skin lesions, as well as reduced proteinuria and glomerular injury; MIF deficiency was associated with almost complete protection from crescentic nephritis in this model (114). Complementary to this study, treatment of either MRL/*lpr* mice and another lupus-prone strain, NZB/NZW F1 mice, with the MIF antagonist ISO-1, reduced functional and histological indices of glomerulonephritis, inhibited CD74^+^ and CXCR4^+^ leukocyte recruitment, and lowered levels of circulating TNF-α in MRL/*lpr* mice and CCL2 in NZB/NZW F1 mice (115). Expression of mRNA for TNF-α, IL-1β, and CCL2 in kidney tissue was reduced in both strains of lupus-prone mice following treatment with ISO-1. Neither autoantibody production nor T and B cell activation was significantly affected (115), suggesting that the protective effect of MIF inhibition in SLE is dependent on the regulation of innate inflammation rather that autoimmunity. This conclusion is aligned with findings in MIF-deficient MRL/*lpr* mice, in which protection from renal damage was not accompanied by any change in systemic autoantibody levels or local autoantibody deposition (114). In humans, serum MIF levels are increased in patients with SLE, although this can be partly explained by increased GC use (112). However, independent of GC-induced MIF, high serum levels have been positively associated with SLE disease damage (SLICC/ACR index) (112).

To date, few studies have comprehensively investigated the role of MIF polymorphisms in the susceptibility and severity of SLE, thus leaving many questions to be answered as to how *MIF* alleles contribute to pathogenesis. Findings from one study with a multinational cohort of 1369 SLE patients showed that both Caucasian and African-American patients with the high expression haplotype −794 CATT_7_/−173*C had a lower incidence of SLE with higher levels of circulating MIF (37). Moreover, when they looked at the relationship between *MIF* alleles and anti-nuclear antibody (ANA) status, both healthy controls and SLE patients with the high expression CATT_7_ or −173*C alleles or the CATT_7_/−173*C haplotype were less likely to be ANA positive. These findings possibly suggest that high expression *MIF* alleles confer some protection from autoimmunity in SLE. One possible explanation for this is that high expression MIF alleles confer protection against infections, such as community-acquired pneumonia (18), which may be mechanistic triggers for SLE through antigenic mimicry. Conversely, patients within the cohort, who had established SLE with end-organ complications, such as serositis, nephritis, and cerebritis, had lower frequencies of the low expression *MIF* −794 CATT_5_ allele (37). This would suggest that, in patients with established disease, higher levels of MIF are associated with greater pathology, a finding consistent with the murine studies described above.

In contrast to this study, a report on a Mexican SLE cohort showed both the −173*C and −794 CATT_7_ polymorphisms increased susceptibility to SLE (35). In this study, both serum MIF and TNF-α were significantly increased in SLE patients and in patients with the high expression polymorphisms. Similarly, Sánchez et al. (36), reported that the −173*C allele was associated with increased susceptibility to SLE in a Spanish cohort and that homozygosity (−173C/C) increased susceptibility further. Moreover, the −173*C haplotype with the −794 CATT_7_ allele conferred a twofold increase in susceptibility to SLE. In this study, none of the MIF polymorphisms were significantly associated with specific clinical manifestations.

## MIF as a Potential Therapeutic Target

In healthy individuals, MIF is typically found circulating in plasma at a range between 2 and 8 ng/ml. However, in autoimmune disease MIF concentrations can fluctuate to markedly higher levels. As such, MIF is commonly seen as a hallmark of disease progression and chronicity, even if increased levels of MIF are the consequence of exacerbated inflammatory cascades, rather than a primary cause of disease (116). By virtue of its breadth of activities, MIF is an essential regulator of innate and inflammatory responses (11). Conversely, MIF can also regulate physiological cell activities enzymatically, as a d-dopachrome tautomerase, phenylpyruvate tautomerase, or a thiol-protein oxidoreductase (14, 117, 118). Given MIF is a pluripotent protein with a range of biological functions, it has become an attractive small molecule and antibody target for therapeutic intervention in autoimmune inflammatory disorders. Currently, there are several classes of small molecule inhibitors of MIF that are designed to interact with MIF at its tautomerase active site and attenuate its pro-inflammatory activities. Most of these inhibitors, including ISO-1 and related molecules, work through direct binding to the active site, allosteric inhibition, modification of residues within the active site or disturbance to the tautomerase trimer (119–121). However, the majority of the reported compounds are not suitable candidates for pharmaceutical development due to the high concentrations (micromolar) required for activity (122). Currently, there are 11 classes of MIF small molecule inhibitors described within the literature [previously comprehensively reviewed in Ref. (123)]; one recently reported novel compound class was shown to have protective effects in a model of myocardial infarction (124), which is the most common cause of death in SLE patients (125).

To date, only a handful of MIF inhibitors have been found to disrupt MIF-CD74 interactions with IC_50_ values of less than 5 μM (120). More promisingly, AV411 (ibudilast; 3-isobutyryl-2-isopropylpyrazolo-[1,5-a]pyridine), is a non-selective inhibitor of phosphodiesterases that is used clinically as an anti-inflammatory drug to treat bronchial asthma and post-stroke complications. Cho and colleagues demonstrated that AV411 was able to allosterically inhibit MIF’s catalytic capabilities *in vitro* via conversion/substitution of a methyl-group to an amine group, which induces conformational changes in pockets adjacent to the active site (126). Furthermore, AV411 was shown to significantly inhibit chemotactic capabilities of PBMCs at clinically relevant concentrations (10 nM) (127).

Migration inhibitory factor is highly stable in its trimer conformation, but relatively unstable as a monomer. Ebselen, a compound known for its anti-inflammatory and anti-oxidant properties, was reported as the first small molecule inhibitor to interfere with MIF oligomerization through interactions with cysteine residues (119). This results in changes to the structural conformation of MIF, consequently inhibiting its ability to induce AKT phosphorylation and induction of pro-inflammatory cytokines. Furthermore, Ebselen was shown to reduce chemotactic activities of epithelial progenitor cells in the presence of recombinant MIF. More recently, Bai and colleagues have reported on a novel allosteric MIF inhibitor, p425, which occupies the interface of two MIF trimers (128). p425 was shown not only to potently inhibit MIF’s ability to tautomerize 4-hyrdoxy-phenyl pyruvate but also block the interaction between MIF and CD74, thereby hampering its pro-inflammatory actions (128). Molecular docking and modeling techniques have also been extensively used to characterize potential compounds that will specifically interact with MIF to block its tautomerase activities. Most compounds generated following computational analysis are tested *in vitro* using a variety of cell-based assays. However, very few of these *in silico* modeled compounds have been successfully translated for clinical use, due to very high IC_50_ values required for desired inhibitory effects (120, 129, 130). It is important to note that the connection, if any, between MIF’s tautomerase activity and pro-inflammatory actions, is unclear [see Ref. (131)]. Drugs which inhibit tautomerase activity might also induce conformational changes that alter MIF signaling and *vice versa*.

The use of anti-MIF neutralizing antibodies has be shown to be therapeutically efficacious in several models of inflammatory and autoimmune diseases (82, 132–135). In a recent study, Tarasuk and colleagues employed the human single-chain variable fragment (HuScFv) monoclonal antibody no. 22 to illustrate binding capabilities specific to MIF using a variety of *in vitro* based assays (136). Moreover, the tautomerase activity of MIF was dose-dependently reduced in the presence of HuScFv antibody through binding of the antibody to catalyitic residues within the tautomerase active site (136). Anti-MIF monoclonal antibody therapy is currently in phase I trials both for solid tumors (NCT01765790) and for lupus nephritis (NCT01541670).

## Future Perspectives

Given the abundance of studies implicating MIF as a fundamental participant in the pathogenesis and progression in autoimmune diseases, MIF may represent a therapeutic target with untapped potential for benefits in the clinic. The effects of MIF to amplify pathogenic pathways, including cytokine expression, T cell activation, and macrophage function, as well as its effects to hinder the efficacy of GCs, mean that antagonizing MIF could have broad application in immune disease. The development of therapeutics using small molecule inhibitors that abrogate tautomerase activity has been limited, as many reported compound classes are not practical for pharmaceutical development. However, the development of anti-MIF monoclonal antibodies has opened new avenues. Understanding the precise mechanisms by which MIF regulates signaling cascades involved in inflammatory conditions will provide new and important insights into the potential to exploit inhibition of MIF to regulate inflammation and immunopathogenesis in autoimmune disorders.

## Conflict of Interest Statement

The authors declare that the research was conducted in the absence of any commercial or financial relationships that could be construed as a potential conflict of interest.
